# Supplementary material for: Customizing a self-healing soft pump for robot
Source: Nat Commun. 2021 Apr 14;12:2247. doi: 10.1038/s41467-021-22391-x (PMC8046788; doi:10.1038/s41467-021-22391-x)
Supplement: Supplementary file 1 — Supplementary Information [file 41467_2021_22391_MOESM1_ESM.pdf]

Supplementary information

Customizing a self-healing soft pump for robot

Tang et al.

This PDF file includes:

Supplementary Fig. 1. Fabrication process of a soft electronic pump.
Supplementary Fig. 2. Three types of soft electronic pumps.
Supplementary Fig. 3. Bidirectional pumping of a soft electronic pump between two liquid reservoirs.
Supplementary Fig. 4. Numerical simulation of the soft electronic pump.
Supplementary Fig. 5. DMA tests at different time and temperature of self-healed films.
Supplementary Fig. 6. Tensile tests.
Supplementary Fig. 7. Photographs of different soft electronic pumps.
Supplementary Fig. 8. Experimental setups for testing the performances of the soft electronic pump.
Supplementary Fig. 9. Customizable pumpabilities of soft electronic pumps.
Supplementary Fig. 10. HVPC and output performances.
Supplementary Fig. 11. Robotic vehicle.
Supplementary Table 1. Comparison of soft electronic pump and stretchable electrohydrodynamic (EHD) pump.
Supplementary Table 2. Difference of four pumps used in Fig. 3.
Supplementary Table 3. Commercially available pumps and compressors.

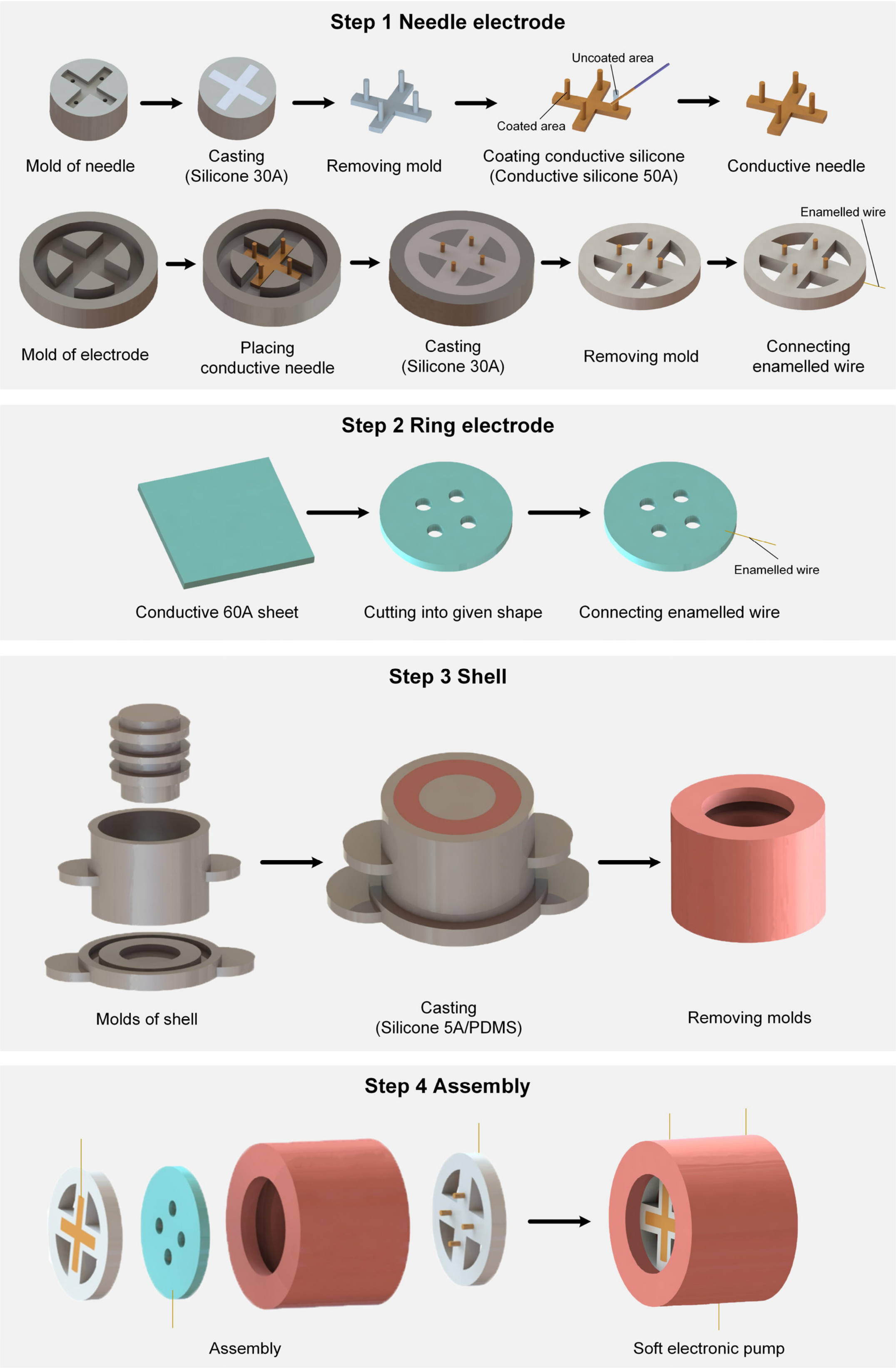

Supplementary Fig. 1 | Fabrication process of a soft electronic pump. The fabrication process mainly includes four steps: (i) Step 1 – Fabrication of needle electrode, (ii) Step 2 – Fabrication of ring electrode, (iii) Step 3 – Fabrication of shell, and (iv) Step 4 – Assembly of all components. All parts of the soft electronic pump are soft and stretchable, making pump fully soft.

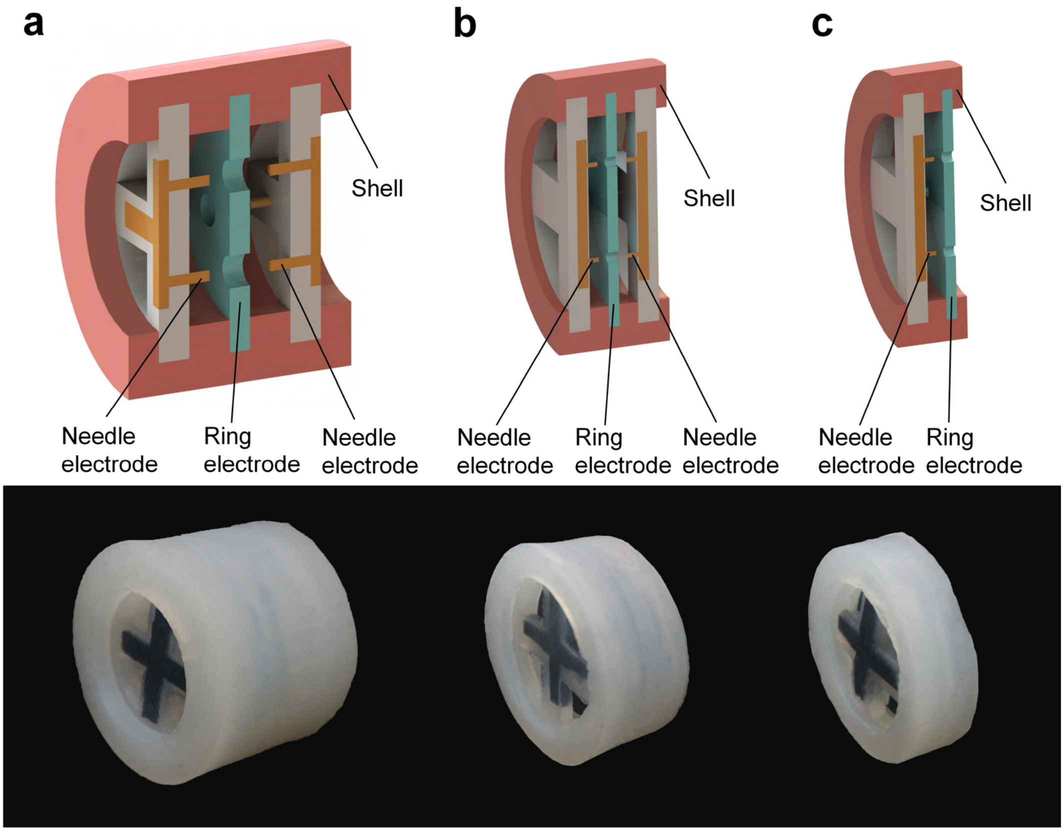

Supplementary Fig. 2 | Three types of soft electronic pumps. a, Type 1. The inner diameter, outer diameter, and length of the shell are 1.8 cm, 3.0 cm, and 2.2 cm, respectively. The diameter of needle, the diameter of hole, and the electrode gap are 1 mm, 3 mm, and 2 mm, respectively. **b**, Type 2. The inner diameter, outer diameter, and length of the shell are 1.8 cm, 2.6 cm, and 1.06 cm, respectively. The diameter of needle, the diameter of hole, and the electrode gap are 0.4 mm, 1 mm, and 0.8 mm, respectively. **c**, Type 3. The inner diameter, outer diameter, and length of the shell are 1.8 cm, 2.6 cm, and 0.68 cm, respectively. The diameter of needle, the diameter of hole, and the electrode gap are 0.4 mm, 1 mm, and 0.8 mm, respectively. The weight of the three types of soft electronic pumps are ~ 13 g, 4.4 g, 3 g, respectively.

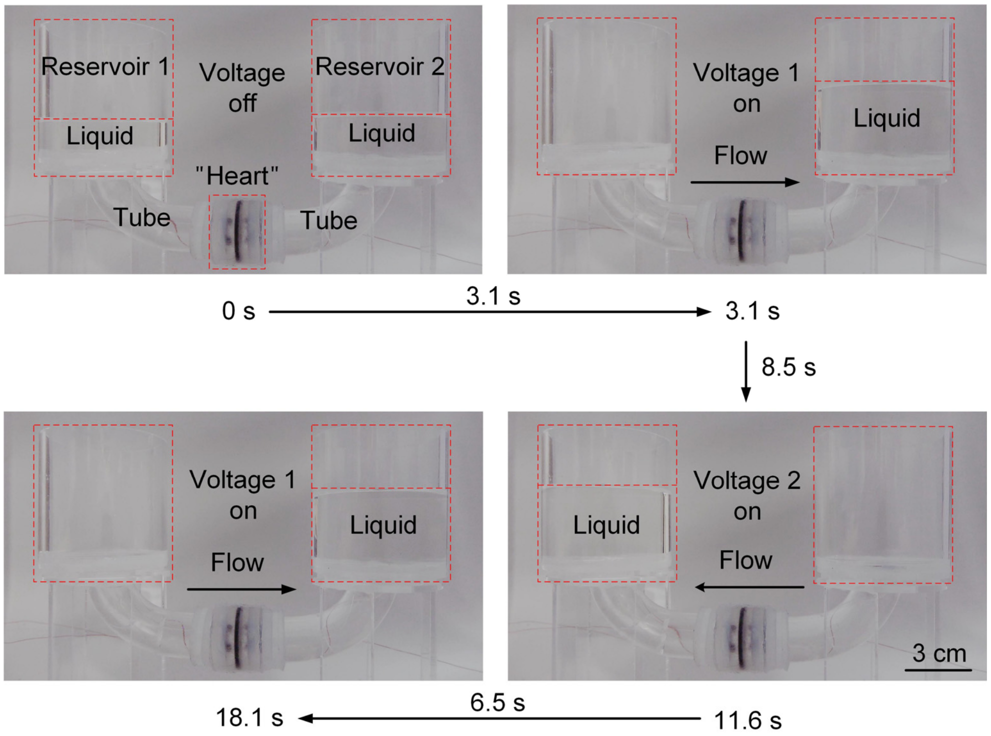

Supplementary Fig. 3 | Bidirectional pumping of a soft electronic pump between two liquid reservoirs. The pumping time between the two cylindrical liquid reservoirs is ~ 3.1 s, 8.5 s, and 6.5 s, respectively, and the total time is ~ 18.1 s under 16 kV.

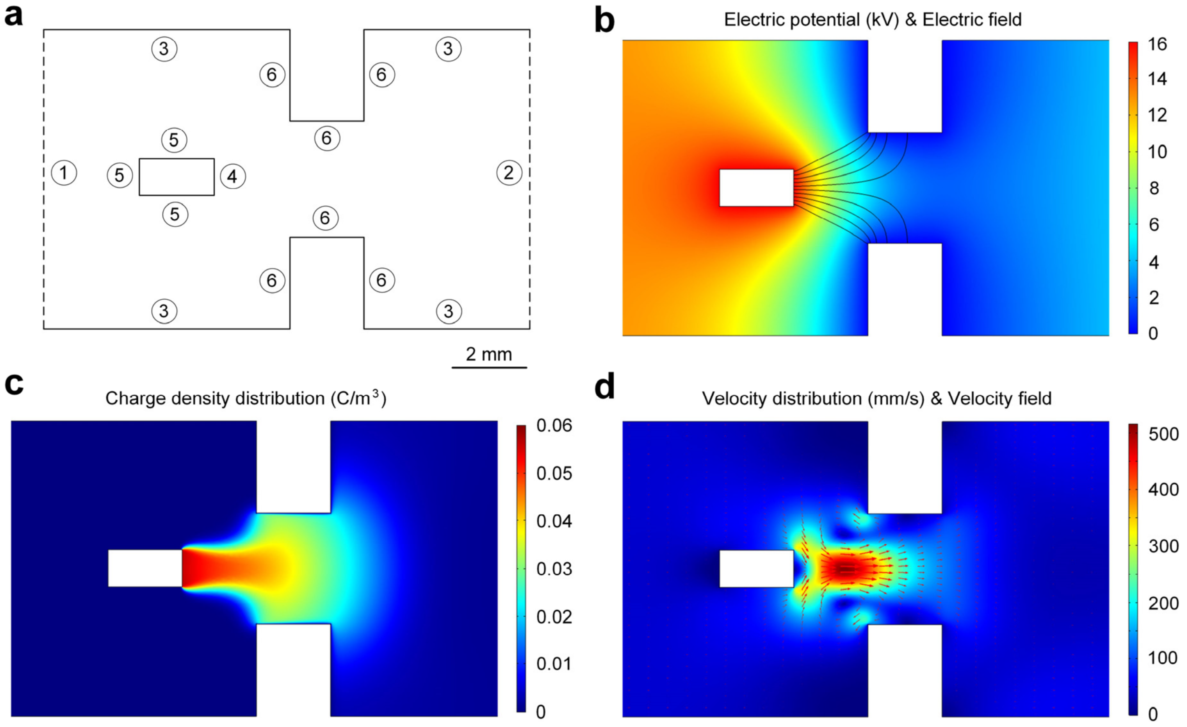

Supplementary Fig. 4 | Numerical simulation of the soft electronic pump. a, Computational setup. **b**, Electric potential distribution and electric field. **c**, Charge density distribution. **d**, Velocity distribution and velocity field.

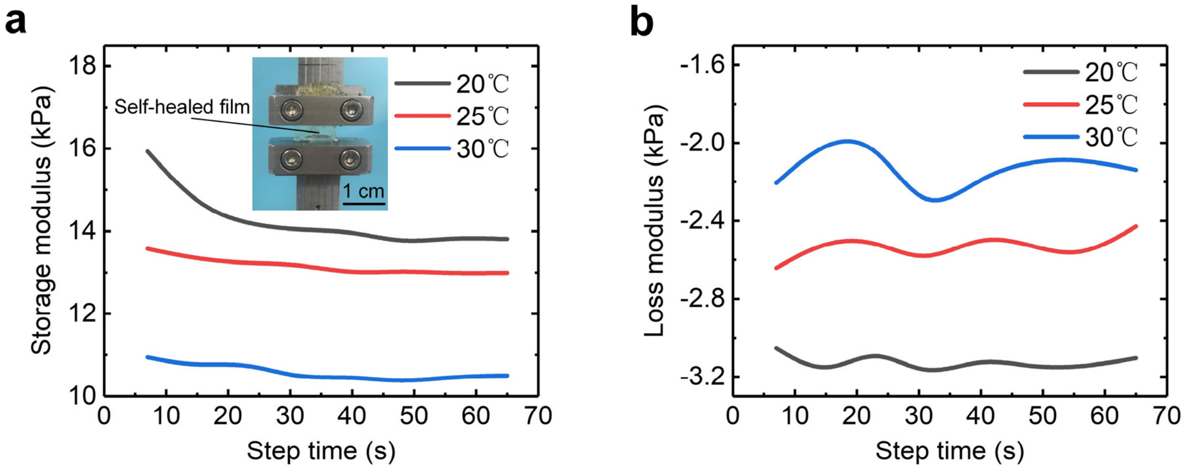

Supplementary Fig. 5 | DMA tests at different time and temperature of self-healed films. **a**, Storage modulus of self-healed films in 20 °C, 25 °C, and 30 °C. **b**, Loss modulus of self-healed films in 20 °C, 25 °C, and 30 °C.

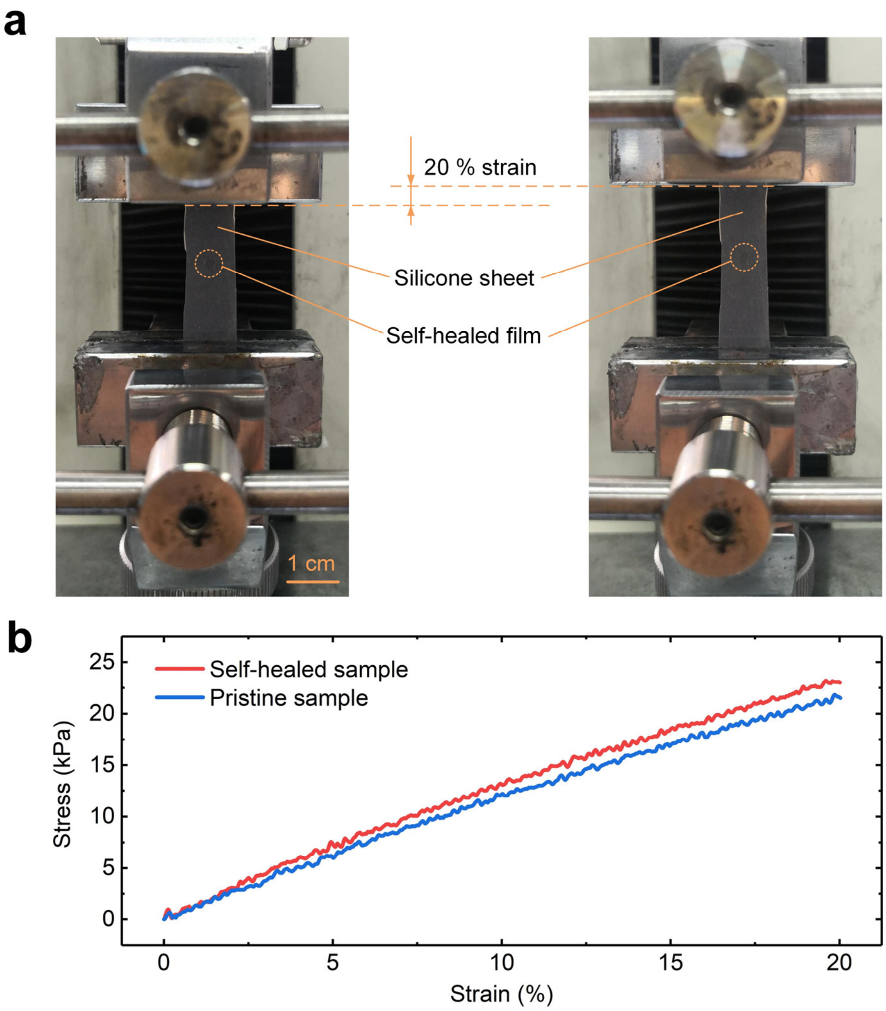

Supplementary Fig. 6 | Tensile tests. **a**, Tensile test of a silicone sheet bonding with a self-healed film. **b**, Tensile stress-strain curves of pristine and self-healed samples.

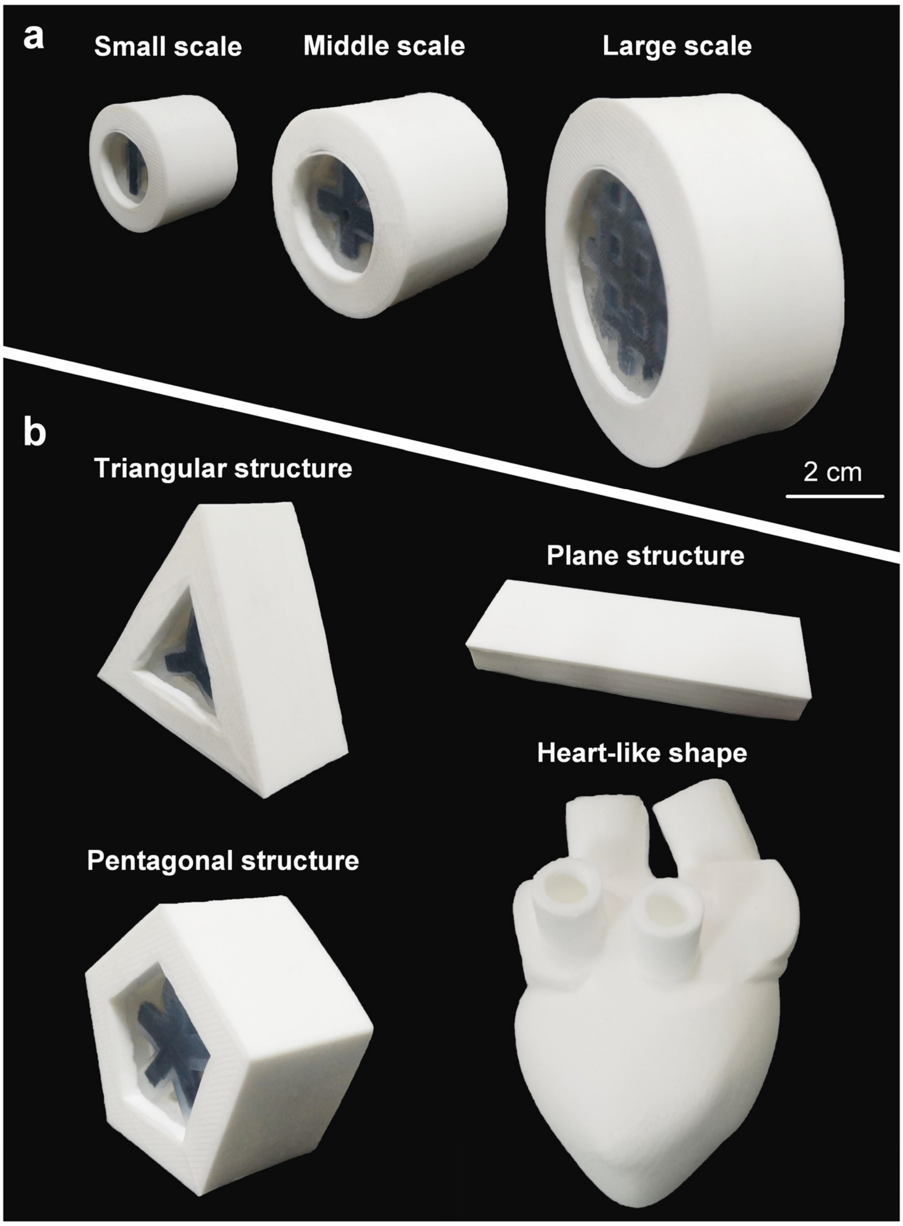

Supplementary Fig. 7 | Photographs of different soft electronic pumps. The shells of the pumps are fabricated by 3D printing using thermoplastic elastomer (TPE) with a shore hardness 80 A.

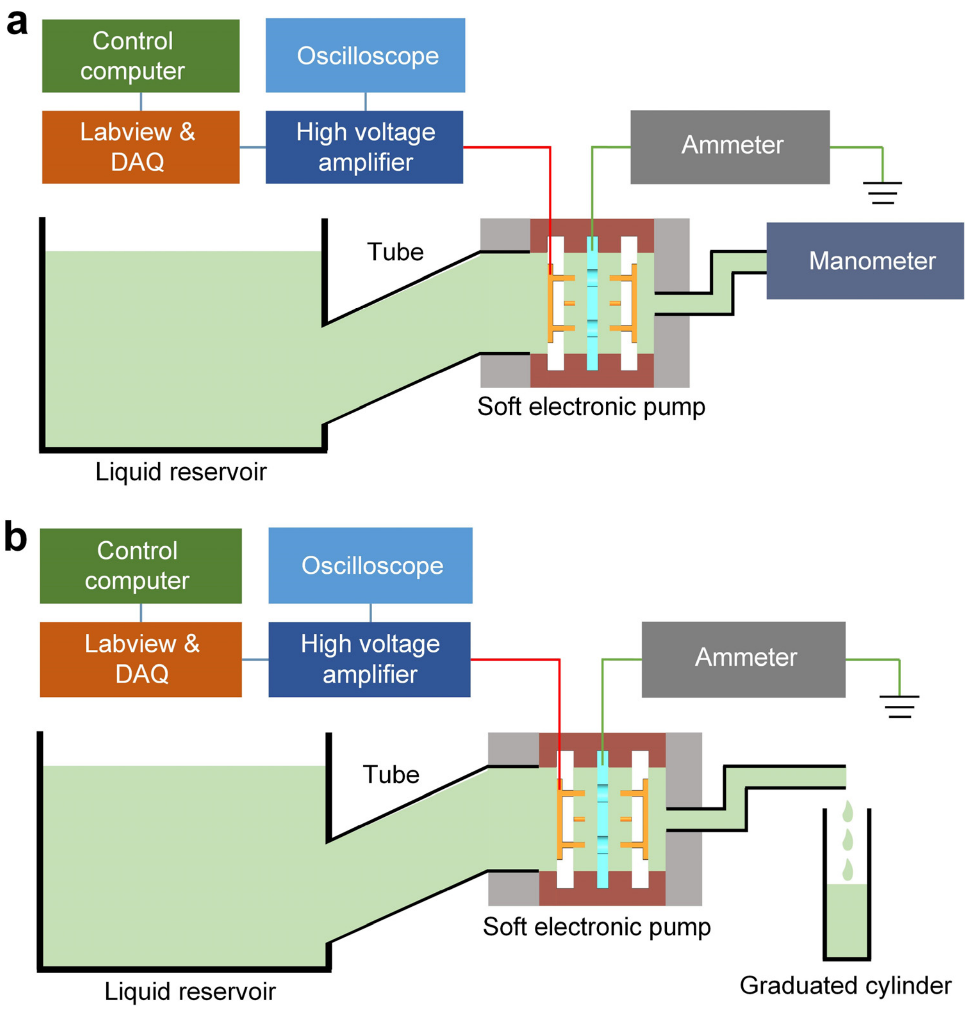

Supplementary Fig. 8 | Experimental setups for testing the performances of the soft electronic pump. a, Experimental setup for testing generated pressures. A custom LabVIEW program, a data acquisition board, and a voltage amplifier are combined to generate the high voltage. An oscilloscope is used to display the high voltage signals. **b**, Experimental setup for testing flow rates.

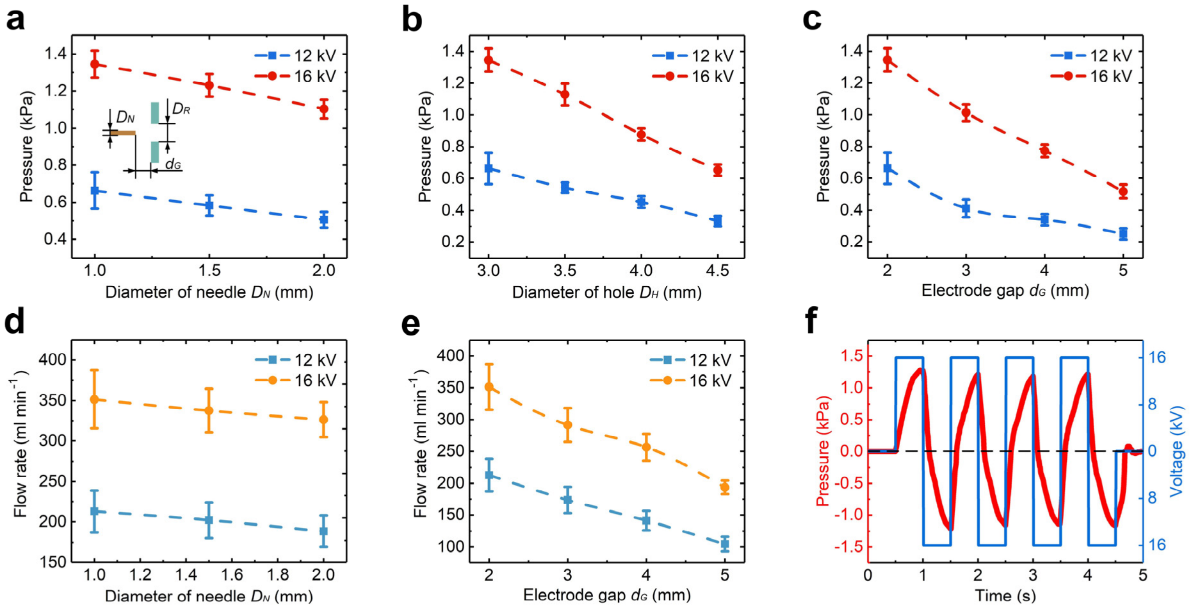

Supplementary Fig. 9 | Customizable pumpabilities of soft electronic pumps. a, Influence of the diameter of needle (D_N) on the generated pressure. **b**, Influence of the diameter of hole (D_H) on the generated pressure. **c**, Influence of the electrode gap (d_G) on the generated pressure. **d**, Influence of the diameter of needle (D_N) on the flow rate. **e**, Influence of the electrode gap (d_G) on the flow rate. **f**, Dynamic generated pressure of soft electronic pumps under the applied square wave of 16-kV amplitude and 1-Hz frequency.

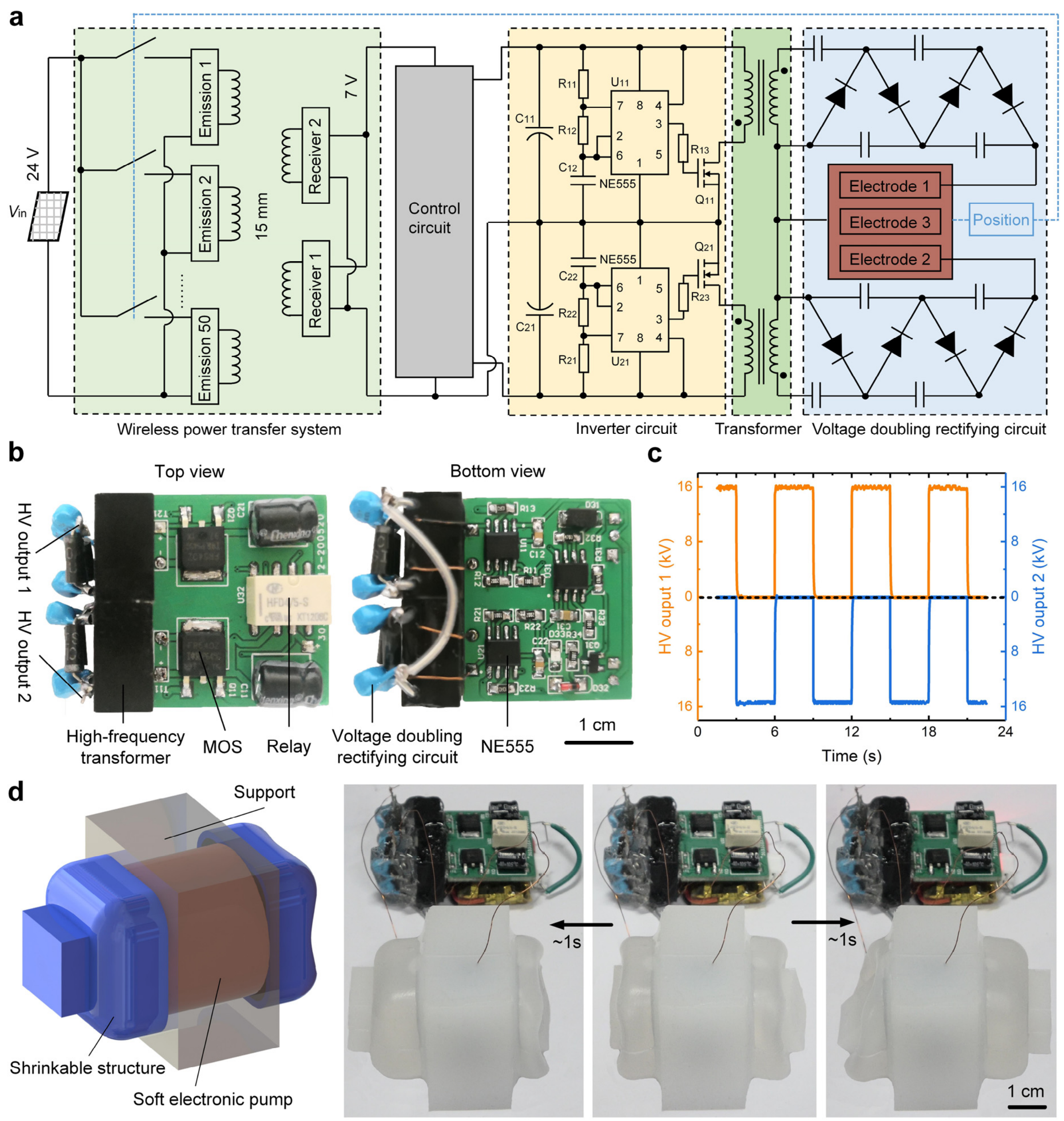

Supplementary Fig. 10 | HVPC and output performances. a, Architecture of the HVPC powered by wireless power transfer system. The circuit consists of four parts: a wireless power transfer system that transfers 24 V power to 7 V through a wireless distance of 15 mm; an inverter circuit that converts 7 V direct current to an intermittent high-frequency square wave pulse; a high-frequency transformer that boosts the alternating-current voltage; and a voltage doubling rectifying circuit that rectifies the high-frequency alternating current into intermittent direct current (square wave). **b**, Image of the HVPC. The weights of the HVPC without encapsulation and with encapsulation are ~16.8 g and ~19 g, respectively. The dimensions of the HVPC are about 40 mm × 30 mm × 16 mm. **c**, Square wave output voltage of the HVPC with frequency of 1/6 Hz. **d**, Design and actuation of the soft bidirectional actuator implanted into a soft electronic pump. Under the power supply of the HVPC, the soft bidirectional actuator achieves rapid bidirectional motion, and the response time of the actuator is ~ 1 s.

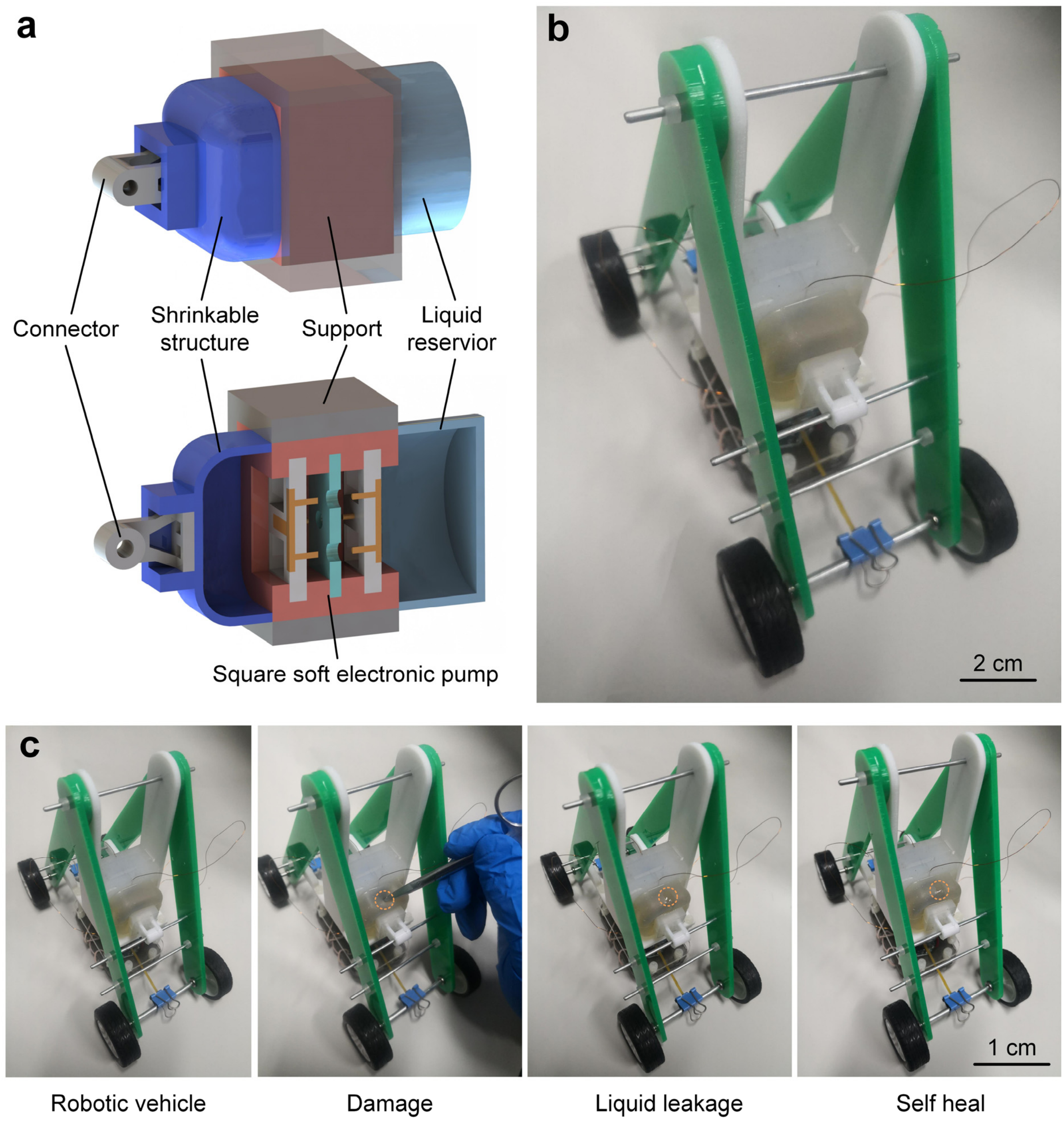

Supplementary Fig. 11 | Robotic vehicle. a, Design of the soft linear actuator implanted into a square soft electronic pump. **b**, Image of the robotic vehicle. **c**, Self-healing process of the robotic vehicle.

	**Soft electronic pump**	Stretchable EHD pump[1]
Underlying mechanism	Positive ions migration	Negative ions migration
Electrode structure	Needle-hole electrode pair (spatial structure)	Plane electrode pair (plane structure)
Migration ion species	Positive ions	Negative ions
Speed of ion migration	Positive ions > Negative ions	
Flow channel	Large cross-areas	Long and narrow channels
Flow direction	Positive electrode to grounding electrode	Grounding electrode to positive electrode
Number of electrode pairs in current version	4	34
Pressure of single electrode pair	~ 9.2 kPa	~ 14 kPa/34 = ~ 0.41 kPa
Flow rate	~ 521 ml min^{-1}	~ 100 μl s^{-1} = 6 ml min^{-1}
System response speed when the pumps are embedded into actuators	~ 1 s	~ 30 s
Electrode materials	Conductive silicone materials long lifetime + good stretchability	C or Ag electrodes C – short lifetime (only 15 min) Ag – poor stretchability
Appearance	Arbitrary	Plane structure

Supplementary Table 1 | Comparison of soft electronic pump and stretchable electrohydrodynamic (EHD) pump.

	Electrode configuration	Functional liquid
**Pump 1**	Electrode design 1	Liquid 1
**Pump 2**	Electrode design 1	Liquid 2
**Pump 3**	Electrode design 2	Liquid 1
**Pump 4**	Electrode design 2	Liquid 2

Note: Electrode design 1 - 1-mm diameter needle, 3-mm diameter hole, and 2-mm gap between them; Electrode design 2 - 0.4-mm diameter needle, 1-mm diameter hole, and 0.8-mm gap between them; Liquid 1 - Dibutyl sebacate based functional liquid; Liquid 2 - Linalyl acetate based functional liquid

Supplementary Table 2 | Difference of four pumps used in Fig. 3. These four pumps are a combination of two kinds of electrode designs and two kinds of liquids.

	Kamoer, EDLP600[2]	SKOOCOM, SC3711PW[3]	SYDSCI, DLP100-DC[4]	EASYWAY, E8L-550W[5]	OUTSTANDING, 750-30L[6]
Power	5 W	8 W	2.5 W	550 W	750 W
Diameter	2.7 cm	2.7 cm	2.7 cm	30 cm	30 cm
Length	6.2 cm	6.1 cm	5.2 cm	54 cm	63 cm
Weight	65 g	60 g	105 g	19 kg	19.5 kg
Pressure	50 kPa	70 kPa	75 kPa	700 kPa	700 kPa
Flow rate	0.6 L min^{-1}	1.5 L min^{-1}	0.3 L min^{-1}	40 L min^{-1}	60 L min^{-1}
Specific pressure	769.23 kPa kg^{-1}	1166.67 kPa kg^{-1}	714.29 kPa kg^{-1}	36.84 kPa kg^{-1}	35.90 kPa kg^{-1}
Specific flow rate	9230.77 ml min^{-1} kg^{-1}	25000.00 ml min^{-1} kg^{-1}	2857.14 ml min^{-1} kg^{-1}	2105.26 ml min^{-1} kg^{-1}	3076.92 ml min^{-1} kg^{-1}

Supplementary Table 3 | Commercially available pumps and compressors.

Supplementary References

1. Cacucciolo, V. et al. Stretchable pumps for soft machines. *Nature* **572**, 516–519 (2019).
2. Kamoer, EDLP600. http://www.kamoer.com/product/product546.html.
3. SKOOCOM, SC3711PW. http://www.skoocomtech.com/water-pump/food-grade-water-pump-sc3711pw.html.
4. SYDSCI, DLP100-DC. https://item.taobao.com/item.htm?spm=a1z10.5-c.w4002-12758764681.19.58047364TEm1Wf&id=565625449977.
5. EASYWAY, E8L-550W. https://detail.tmall.com/item.htm?spm=a230r.1.14.194.57b752f6DwJSCk&id=616890263692&ns=1&abbucket=1.
6. OUTSTANDING, 750-30L. https://detail.tmall.com/item.htm?spm=a230r.1.14.89.57b752f6DwJSCk&id=42490750769&ns=1&abbucket=1.
