# Supplementary material for: Customizing a self-healing soft pump for robot
Source: Nat Commun. 2021 Apr 14;12:2247. doi: 10.1038/s41467-021-22391-x (PMC8046788; doi:10.1038/s41467-021-22391-x)
Supplement: Supplementary file 3 — Description Of Additional Supplementary Files [file 41467_2021_22391_MOESM3_ESM.docx]

**Description of Additional Supplementary Files**

File Name: Supplementary Video 1: Assembly of soft electronic pump

Description: This video shows the softness of the parts (including shell, needle electrode, and ring electrode) and the process of assembly, illustrating that the soft electronic pump is fully soft and stretchable.

File Name: Supplementary Video 2: Pumping of soft electronic pump

Description: A soft electronic pump is connected to two cylindrical liquid reservoirs with connectors and tubes so as to pump liquid between them. The shell of the pump is made of PDMS, making it transparent. The applied voltage signal is a switching square wave with three different amplitudes of 16 kV, 12 kV, and 8 kV. This video illustrates that the soft electronic pump possesses rapid, controllable, and fast-switching bidirectional pumping capacity.

File Name: Supplementary Video 3: Self-healing of soft electronic pump

Description: The fluidic system consists of a body and a soft electronic pump, and filled with self-healing liquid. The body is made of Ecoflex 00-20. The applied voltage signals are a 16-kV DC voltage and a 16-kV square wave. The soft electronic pump pumps liquid out from the damage when the fluidic system is punctured, and then the liquid heals the damage when the fluidic system is placed uprightly for ~ 6 hours at ambient temperature (35 °C). This video illustrates that the soft electronic pump is capable of pumping self-healing liquid to power robotic system and heal the damage of the system.

File Name: Supplementary Video 4: A soft bidirectional actuator powered by a soft electronic pump

Description: The soft electronic pump is implanted into a soft-matter structure to form a self-contained soft bidirectional actuator. Under the power supply of the HVPC, the soft bidirectional actuator achieves rapid bidirectional motion, and the response time of the actuator is ~ 1 s.

File Name: Supplementary Video 5: A soft robotic fish powered by a circular soft electronic pump

Description: The circular soft electronic pump is implanted into a soft robotic fish, driving a bidirectional two-chamber bending actuator to achieve swing motion of tail fin. Under the power supply of the HVPC with a lithium battery, the soft robotic fish achieves silent and untethered swimming motion underwater.

File Name: Supplementary Video 6: A robotic vehicle powered by a square soft electronic pump

Description: The square soft electronic pump is implanted into a robotic vehicle, driving a linear actuator to actuate the robotic vehicle. Under the power supply of the HVPC with two receiving coils, the robotic vehicle moves forward powered by the wireless power transfer system.
